# The Gut Microbiome and Metabolome of Two Riparian Communities in the Amazon

**DOI:** 10.3389/fmicb.2019.02003

**Published:** 2019-09-04

**Authors:** Eder Soares Pires, Cristiane Cassiolato Pires Hardoim, Karla Rodrigues Miranda, Danielle Angst Secco, Leandro Araújo Lobo, Denise Pires de Carvalho, Jun Han, Christoph H. Borchers, Rosana B. R. Ferreira, Joana Falcão Salles, Regina Maria Cavalcanti Pilotto Domingues, Luis Caetano Martha Antunes

**Affiliations:** ^1^Instituto de Microbiologia Paulo de Góes, Universidade Federal do Rio de Janeiro, Rio de Janeiro, Brazil; ^2^Instituto Tecnológico Vale – Desenvolvimento Sustentável, Belém, Brazil; ^3^Instituto de Biociências, Universidade Estadual Paulista, São Vicente, Brazil; ^4^Instituto de Biofísica Carlos Chagas Filho, Universidade Federal do Rio de Janeiro, aRio de Janeiro, Brazil; ^5^University of Victoria – Genome British Columbia Proteomics Centre, University of Victoria, Victoria, BC, Canada; ^6^Department of Biochemistry and Microbiology, University of Victoria, Victoria, BC, Canada; ^7^Segal Cancer Proteomics Centre, Lady Davis Institute, Jewish General Hospital, McGill University, Montreal, QC, Canada; ^8^Gerald Bronfman Department of Oncology, Jewish General Hospital, McGill University, Montreal, QC, Canada; ^9^Microbial Ecology Cluster, Groningen Institute for Evolutionary Life Sciences, University of Groningen, Groningen, Netherlands; ^10^Escola Nacional de Saúde Pública Sergio Arouca, Fundação Oswaldo Cruz, Rio de Janeiro, Brazil; ^11^Instituto Nacional de Ciência e Tecnologia de Inovação em Doenças de Populações Negligenciadas, Centro de Desenvolvimento Tecnológico em Saúde, Fundação Oswaldo Cruz, Rio de Janeiro, Brazil

**Keywords:** gut microbiome, riparian communities, Amazon, high-throughput sequencing, metabolic prediction, metabolomics

## Abstract

During the last decades it has become increasingly clear that the microbes that live on and in humans are critical for health. The communities they form, termed microbiomes, are involved in fundamental processes such as the maturation and constant regulation of the immune system. Additionally, they constitute a strong defense barrier to invading pathogens, and are also intricately linked to nutrition. The parameters that affect the establishment and maintenance of these microbial communities are diverse, and include the genetic background, mode of birth, nutrition, hygiene, and host lifestyle in general. Here, we describe the characterization of the gut microbiome of individuals living in the Amazon, and the comparison of these microbial communities to those found in individuals from an urban, industrialized setting. Our results showed striking differences in microbial communities from these two types of populations. Additionally, we used high-throughput metabolomics to study the chemical ecology of the gut environment and found significant metabolic changes between the two populations. Although we cannot point out a single cause for the microbial and metabolic changes observed between Amazonian and urban individuals, they are likely to include dietary differences as well as diverse patterns of environmental exposure. To our knowledge, this is the first description of gut microbial and metabolic profiles in Amazonian populations, and it provides a starting point for thorough characterizations of the impact of individual environmental conditions on the human microbiome and metabolome.

## Introduction

The gastrointestinal tract harbors most of the human microbiota and is the body site with the highest microbial population density. Although the microbiota is composed of bacteria, fungi and protists, the vast majority of commensal microbes in the human body are bacteria. In fact, it is estimated that fifty percent of the fecal mass is composed of commensal bacteria (Sender et al., 2016). The dominant phyla in the human gut microbiota are *Firmicutes*, *Bacteroidetes*, *Actinobacteria*, and *Proteobacteria*, with other common phyla, such as *Verrucomicrobia* and *Fusobacteria* found as less prevalent components (Sekirov et al., 2010). This commensal bacteriome plays important roles in human health, performing diverse metabolic functions such as the fermentation of fibers, production of vitamins, energy recovery by the absorption of microbe-derived short-chain fatty acids, carbohydrate metabolism, regulation and stimulation of the host immune system, amongst others. It is also a well-established fact that the microbiota plays a crucial role in maintaining the integrity of the intestinal mucosa and protecting its host from invading pathogens (Sekirov et al., 2010).

Within the human gut, the highest density of microbiota is found in the colon. These communities are stable, and it is thought that this stability is a consequence of the co-evolution of the host and the multiple microbial species in this environment, which resulted in the selection of microbial strains that are highly adapted to this particular niche (Huffnagle, 2010). As such, these communities are highly resilient, being capable of adjusting themselves back to their original composition after a disturbance (Allison and Martiny, 2008; Huffnagle, 2010). However, many factors, including stresses like the use of antibiotics and other bioactive compounds can cause significant transient changes in these microbial populations (Dethlefsen et al., 2008; Sekirov et al., 2008; Turnbaugh et al., 2008; Antonopoulos et al., 2009; Bailey et al., 2010, 2011). Of note, it has been shown that the diet can have significant effects on the intestinal microbiota composition (Bezirtzoglou et al., 2011; Kau et al., 2011; Wu et al., 2011). Arumugam et al. (2011) have previously found that bacterial communities from the human gut could be classified into two main groups (enterotypes) based on the relative abundance of the *Bacteroides* and *Prevotella* genera (both within the *Bacteroidetes* phylum). Building up on this observation, Wu et al. (2011) performed a study on the impact of diet on the human gut microbiome and showed that there is a strong association between gut microbiota enterotypes and host diet. Although the categorization of the human gut microbiome in enterotypes has been largely questioned (Jeffery et al., 2012; Costea et al., 2018), these and other studies have provided irrefutable evidence that diet is an important factor in shaping the microbial communities living in the human gut.

From an ecological and nutritional standpoint, the Amazon region and its population are highly unique. In the communities that live along the major riverbanks of the Amazon, the diet is predominantly composed of fish and manioc flour. As the traditional people of this region do not have several of the resources required for productive agriculture, their survival is almost exclusively dependent upon hunting, fishing and collecting wild seeds, such as chestnut. Within the complex environment represented by the Amazon, the local riparian population of the Madeira River is composed mainly of individuals of indigenous descent. However, they were also interbred during the European colonization era, as well as with Northeastern individuals that came during the rubber and gold rushes. These communities along the Madeira River range from small remote villages, with only a few dozen people, to larger municipalities with over 50,000 inhabitants. Many coastal communities prefer to establish their homes near the lakes formed by small rivers and streams, tributaries of major rivers, and these such lakes are very abundant in this region. Therefore, individuals of these riverine communities represent a very distinct group, because they have very special characteristics with regards to their living environment and eating habits.

In order to probe the effects of this environment on the microbial and chemical ecology of the human gut microbiome, we characterized the bacterial composition of feces from individuals from two riverine communities in the Amazon. Additionally, we characterized bacterial populations found in the feces of individuals living in Rio de Janeiro, southeastern Brazil, a highly populated and industrialized city. The lifestyle in Rio de Janeiro is markedly different from that of Amazonian riparian communities, and these samples were therefore used as a contrasting group. Lastly, we investigated the chemical composition of the gut metabolome of these three groups of individuals using high-throughput metabolomics, with the goal of comparing the metabolic activity of their microbiomes. By doing so, we found significant differences in gut bacterial and chemical composition when the two Amazon populations were compared to individuals from the urban environment. On the other hand, no striking differences were found when the gut microbiome and metabolome of the two Amazon populations were compared. To our knowledge, this is the first report of gut microbiome and metabolome investigations of Amazon populations. These data highlight environmental conditions, such as nutrition, as critical factors for the functioning of the human gut microbial ecosystem. Further studies will reveal additional details and specificities of microbiomes from Amazon populations, and their potential impact on health.

## Materials and Methods

### Ethics Statement

This study was approved by the Research Ethics Committee of the Institute for Studies of Collective Health of the Federal University of Rio de Janeiro, under number 48/2011. Written informed consent was obtained from all participants of the study.

### Fecal Samples

Forty-three healthy individuals were recruited for this study. Fifteen subjects were from the Puruzinho Lake community and fifteen were from the Buiuçu community, both in the Amazon. The approximate location of the Puruzinho community is 7^*o*^22′23.0″S 63^*o*^03′00.2″W, and the community has about 120 inhabitants. The Buiuçu community is located approximately at 7^*o*^24′01.9″S 63^*o*^00′22.1″W, and has about 40 inhabitants. In addition to these communities, thirteen individuals from the city of Rio de Janeiro were included in this study. Individuals recruited varied between 19 and 58 years of age. Fifteen subjects (34.9%) were male and 28 (65.1%) were female. Subject characteristics are shown in Table 1. Fresh fecal samples were collected by each individual and placed in their household freezer immediately after collection. Samples were picked up the following day, and kept frozen until they arrived in the laboratory, where they were stored at −80°C until used for DNA and metabolite extractions.

**TABLE 1:** Characteristics of subjects from the three communities studied.

**Community**	**Number and gender^a^**	**Age range (median)^b^**
Buiuçu	9F, 6M	20–44 (28)
Puruzinho	9F, 6M	23–58 (35)
Rio de Janeiro	10F, 3M	19–49 (27)

*^*a*^F-female; M-male. ^*b*^In years.*

### DNA Extraction

DNA extraction was performed using the QIAamp^®^ DNA Stool Mini Kit (Qiagen, Dusseldorf, Germany) following the manufacturer’s instructions and with an additional step for mechanical bacterial lysis. Approximately 200 mg of feces were used for DNA extraction. DNA concentrations were determined using a Qubit fluorometer (Invitrogen, Waltham, MA, United States).

### 16S rDNA Illumina Sequencing

The V4 region of the 16S ribosomal RNA (rRNA) gene was amplified using primers 515F (5′-GTGYCAGCMGCCGC GGTAA-3′) and 806R (5′-GGACTACNVGGGTWTCTAAT-3′) (Apprill et al., 2015), 100–150 ng of template DNA, 1.5 mM MgCl_2_, 1× PCR buffer, 0.8 mM of dNTPs (dATP, dCTP, dGTP, dTTP), 0.32 μM of each primer and 2 U of GoTaq^®^ DNA Polymerase (Promega, Madison, WI, United States) in 50 μL. Cycling was performed as follows: one denaturing cycle at 98°C for 2 min, followed by 30 cycles at 98°C for 45 s, 56°C for 1 min and 72°C for 90 s, followed by a final cycle at 72°C for 10 min. Amplicons were analyzed in 1.5% agarose gels stained with 0.5 μg/mL of ethidium bromide in TAE buffer (40 mM Tris-acetate and 1 mM EDTA, pH 8.2) under a constant voltage of 100 V. Bands were excised and extracted from gels using Ultrafree-DA Centrifugal Filter Units (Millipore, Darmstadt, Germany). Fifty nanograms of the purified material was then used as template on a second PCR to add sequencing barcodes, using the Next Flex 16S V4 Amplicon-Seq Kit for Illumina Platforms (Bioo Scientific, Austin, TX, United States). The 50-μL reaction contained 50 ng of DNA, NEXTflexTM DNA PCR Master Mix, barcoded reverse primers for each sample (NEXTflexTM 16SV4R, 0.2 μM) and a common forward primer (NEXTflexTM 16SV4F, 0.2 μM, 5′-AATGATACGGCGACCACCGAGATCTACACTGTAATTGTG TGCCAGCMGCCGCGTAA-3′). Cycling was performed exactly as described above. The fragments obtained were then purified using the Agencourt AMPure XP kit (Beckman Coulter, Indianapolis, IN, United States) and sent to the Argonne National Laboratory^1^ (Lemont, IL, United States), where they were sequenced using the Illumina MiSeq platform (paired-end 2 × 150-bp sequencing, Illumina, San Diego, CA, United States) according to the manufacturer’s instructions. Sequencing data were deposited in the National Center for Biotechnology Information (NCBI) Sequence Read Archive (SRA) under the accession number PRJNA547608.

### rDNA Sequence Analyses

Sequences were first analyzed using the Quantitative Insights Into Microbial Ecology software package (QIIME) (Caporaso et al., 2010b). First, sequences were separated based on their barcodes and trimmed to remove primer and barcode sequences. Sequences were also subjected to a quality check, and those with quality values below 20 (Phred < Q20) were discarded. The average length of the remaining sequences was 253 bp. Combined sequences produced by alignments of sequences from both strands were then searched against the Greengenes database using PyNAST (Caporaso et al., 2010a). Valid sequences were classified into operational taxonomic units (OTUs) using 97% identity as the threshold for classification using the UCLUST method (Edgar, 2010). Bacteria were classified according to the nearest taxon, depending on the similarity values obtained, using the following cut-off values: >97% for species classification, 95–97% for genera, 90–95% for families, 85–90% for orders, 80–85% for classes and 77–80% for phyla (Ishak et al., 2011). Based on the taxonomy assignment, OTUs classified as Archaea, Chloroplast, Mitochondria and unassigned were removed from the OTU table. Alpha diversity [Shannon-Wiener diversity measures (Shannon, 1948)] was then estimated on rarefied tables at a threshold of 43,000 sequences per sample. Beta diversity was measured using unweighted UniFrac on the rarefied tables, and Principal Coordinate Analysis (PCoA) was performed with unweighted UniFrac distances using the online software MicrobiomeAnalyst^2^. For these analyses, we did not use the Minimum Count or Low Variance filters, and data was not scaled or transformed. Venn diagrams were also constructed to compare OTU composition between different sample groups.

### High-Throughput Metabolomics

Fecal samples were extracted in essentially the same way as previously described (Antunes et al., 2011a, b). Briefly, a 220–320 mg aliquot of feces (wet weight) was added to a 2-mL polypropylene tube and 600 μL of LC/MS-grade acetonitrile (Sigma-Aldrich, Darmstadt, Germany) was added. Samples were thoroughly vortex-mixed and then centrifuged at >21,000 × *g* in a microtube centrifuge (Eppendorf, Hamburg, Germany). Supernatants were collected and 400 μL was transferred to a new tube and dried at room temperature using a speed-vacuum concentrator. A control tube containing the same volume of solvent was used as a background signal control (see below). Extracts were then analyzed by mass spectrometry with direct infusion (DI-MS) on a Bruker Apex-Qe 12-Tesla Fourier transform ion cyclotron resonance instrument. For this, dried metabolite extract residues were dissolved in 75% acetonitrile (in water), at 200 μL per 100 mg of the starting material and the resulting solution was vortexed, sonicated for 1 min, and cleared by centrifugation. For positive-ion DI-FTICR-MS, 100 μL of the solution was mixed with 200 μL of an acetonitrile-water-formic acid-Agilent ESI Tuning Mix standard solution (75:25:0.2:1, v/v). The tuning mix (Agilent, Santa Clara, CA, United States) contained several compounds which were used as internal standards for mass calibration. Samples were then infused, using a syringe pump (KDS Scientific, Holliston, MA, United States), into a mass spectrometer equipped with an Apollo II electrospray ionization source, a quadrupole mass filter, and a hexapole collision cell (Bruker Daltonics, Billerica, MA, United States) at a flow rate of 2.5 μL per minute. Mass spectra were acquired in the positive ESI mode within an *m/z* range of 180 to 1000. For negative-ion DI-FTICR-MS, 100 μL was mixed with 200 μL of acetonitrile-water-ammonium hydroxide-Agilent ESI Tuning Mix standard solution (75:25:0.2:1, v/v). Samples were then infused into the mass spectrometer. Mass spectra acquired in the negative-ion ESI mode were acquired within an *m/z* range of 180 to 1050. To increase detection sensitivity, survey scan mass spectra in positive- and negative-ion modes were acquired from the accumulation of 300 scans per spectrum.

### Mass Spectrometry Data Analysis

Raw mass spectrometric data files were processed in the following manner: raw mass spectra acquired from each sample group were batch processed using the instrument manufacturer’s data analysis software, Data Analysis, but with an in-house written VBA script to do automatic internal mass calibration with the reference masses of the spiked-in ESI tuning mix. Monoisotopic peaks corresponding to the isotopic pattern distributions were then automatically determined, and those with a signal/noise ratio above three were selected. Peaks from the solvent and instrument background noise were removed. Finally, a two-dimensional data matrix (*m/z* versus relative intensity) was generated for each sample and saved in a format amenable for further data analysis. In order to select metabolite features that were present in a significant number of samples, we first filtered our data set to include only ions that were present in *n*-1 samples of at least one of the three groups studied. In other words, only ions present in at least four samples of at least one of the three sample groups (Puruzinho, Buiuçu and Rio de Janeiro) were used in this analysis. Average intensity values were calculated for each metabolite feature detected in each sample group. Fold-changes were also determined, and statistical analyses were performed using Student’s *t* test. Changes of 2-fold or more with a *p* value lower than 0.05 were considered statistically significant. In addition to evaluating fold-changes in specific metabolites, metabolic signatures of each sample group were also compared through the construction of heat maps, dendrograms and Principal Component Analysis (PCA) plots, using the Statistical Analysis tool of the online software MetaboAnalyst^3^. For these analyses, we did not apply any of the Data Filtering, Sample Normalization, Data Transformation or Data Scaling options, and default options were used for the additional fields. Comparisons were performed using the entire data set or the metabolite features that showed the highest discriminating power, as determined by MetaboAnalyst using ANOVA tests. In order to probe the metabolic pathways represented by the metabolites that showed different levels between the communities studied, we used the software Pathos, an online tool that maps metabolites onto KEGG pathways^4^. For these analyses, we used metabolite features whose levels showed at least 2-fold differences when binary comparisons were performed, and their *m/z* values were searched with a mass tolerance of 3 ppm. For positive ionization mode, adducts considered were (M + H)^+^, (M + Na)^+^, and (M + K)^+^. For negative ionization, we used (M-H)^–^, (M + Na-2H)^–^ and (M + K-2H)^–^.

### Statistical Analyses

Microbiome sequencing (OTU table) data were tested to verify whether they followed Gaussian distribution using both the D’Agostino & Pearson normality test as well as the Shapiro-Wilk normality test. Comparisons of OTU abundance and alpha diversity values between sample groups were performed using the Kruskal-Wallis test with multiple comparison False Discovery Rate (FDR) correction using the two-stage linear step-up procedure of Benjamini, Krieger and Yekutiely. ANOVA was used to determine metabolites with the highest discriminatory power within each sample group, and Student’s *t* tests were used for the comparison of individual metabolite relative levels between sample groups. ANOSIM was used to compare beta diversity on PCoA. Correlation between taxa and sample group was performed using Pearson r as the distance measure. Statistical analyses were performed in R or using GraphPad Prism 5 (GraphPad Software, San Diego, CA, United States), MicrobiomeAnalyst or MetaboAnalyst softwares.

## Results

**FIGURE 1:**
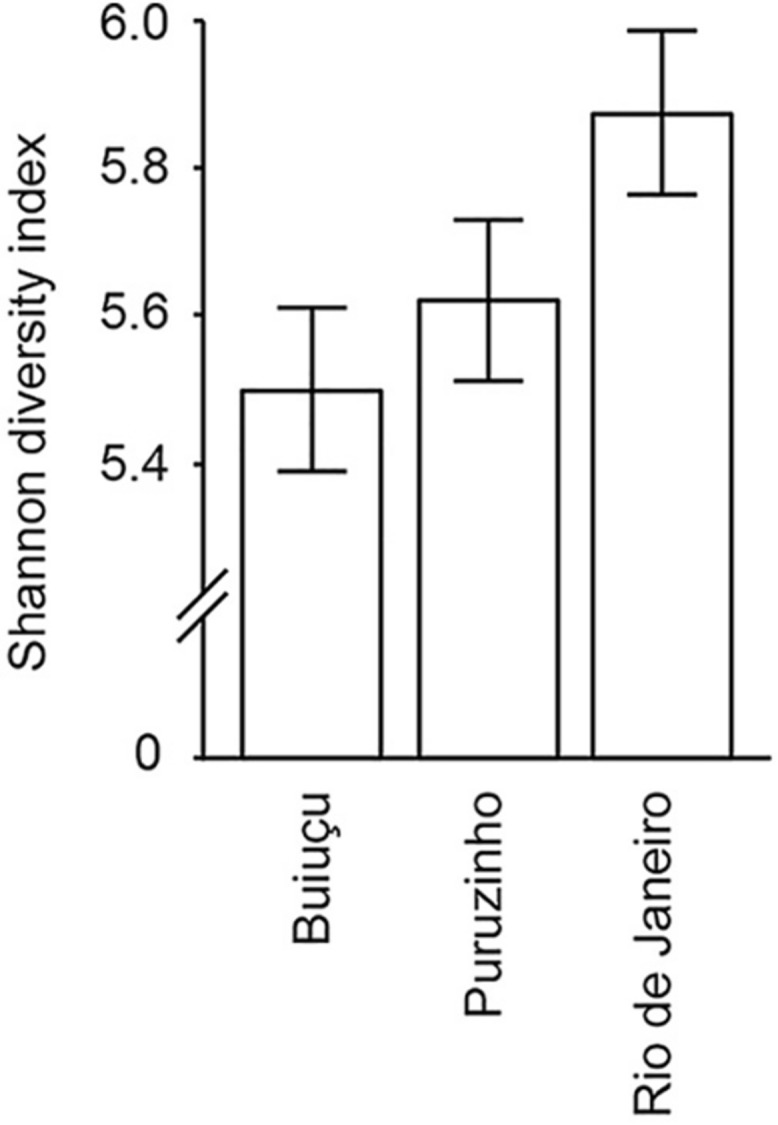
Alpha diversity of the gut microbiome of individuals from the three communities studied. Diversity values and standards deviation are shown, as determined by Shannon index analyses of rarified sequences.

By sequencing 16S rDNA from fecal samples we generated a table containing 1,096 different representative bacterial OTUs, containing a total of 3,007,721 sequences (Supplementary Table S1). The high number of OTUs observed shows the significant bacterial diversity found in the gut of these individuals. Then, alpha diversity was calculated using the Shannon-Wiener diversity measure (Figure 1), and the following values were obtained: Buiuçu, 5.50 ± 0.71; Puruzinho, 5.62 ± 0.81; and Rio de Janeiro, 5.87 ± 0.74. The difference in diversity between the three communities was not significant (*p* = 0.40), as determined by the Kruskal-Wallis test with multiple comparison FDR correction using the Benjamini, Krieger and Yekutiely tests. The most diverse bacterial community was detected in samples from Rio de Janeiro, followed by the Puruzinho and Buiuçu samples.

**FIGURE 2:**
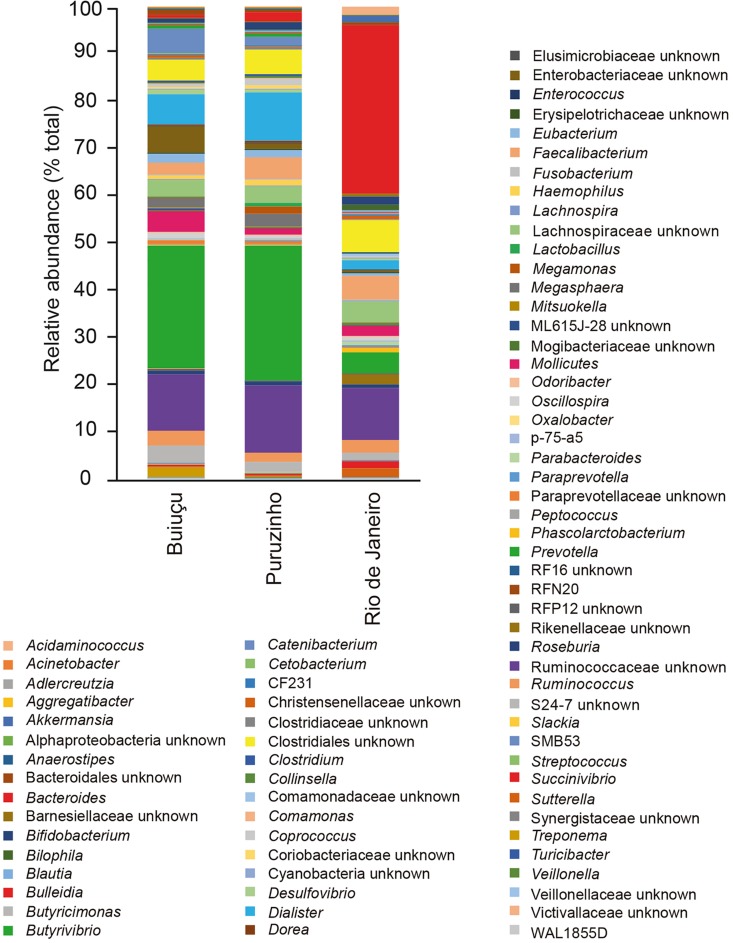
Relative abundance of bacterial genera in the gut microbiome of individuals from the three communities studied.

**FIGURE 3:**
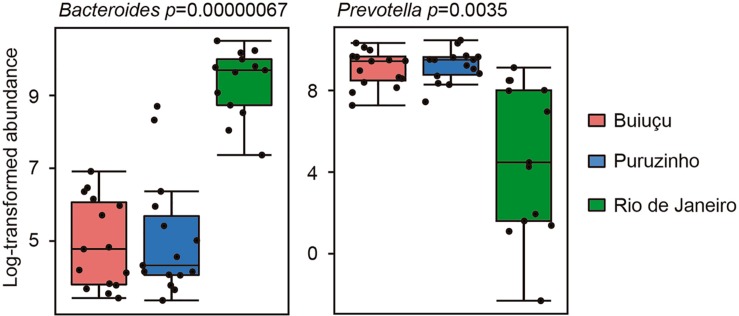
Box plots showing relative abundance of the two bacterial genera showing the highest correlation with the Amazon versus Rio de Janeiro sample groups. Correlation was measured using the Pattern Search tool in MicrobiomeAnalyst, with Pearson r as the distance measure.

Despite the similarity in alpha diversity values, analysis of the relative abundance of representative OTUs indicated differences in the composition of the intestinal bacterial communities among individuals from the three locations. In total, we identified 12 bacterial phyla (*Actinobacteria*, *Bacteroidetes*, *Cyanobacteria*, *Elusimicrobia*, *Firmicutes*, *Fusobacteria*, *Lentisphaerae*, *Proteobacteria*, *Spirochaetes*, *Synergistetes*, *Tenericutes*, *Verrucomicrobia*) in the samples studied, and did not observe significant differences between samples from the three communities (Supplementary Figure S1), as determined by Kruskal-Wallis and Benjamini, Krieger and Yekutiely tests (*p* = 0.88). In our data, the most abundant phyla were *Firmicutes* and *Bacteroidetes*, as expected. For individuals from the Buiuçu community, *Firmicutes* and *Bacteroidetes* encompassed 638 OTUs and 488,769 sequences, and 242 OTUs and 335,195 sequences, respectively (Supplementary Table S2a). *Firmicutes* comprised 624 OTUs and 600,429 sequences, whereas *Bacteroidetes* contained 254 OTUs and 372,699 sequences in samples from the Puruzinho community (Supplementary Table S2b). For the Rio de Janeiro group, *Firmicutes* and *Bacteroidetes* encompassed 617 OTUs and 375,751 sequences, and 270 OTUs and 437,319 sequences, respectively (Supplementary Table S2c). Similarly, when the sequence data obtained was analyzed at the family level, no significant differences in the relative abundance of bacterial families were seen when comparing samples from the three communities, as determined by Kruskal-Wallis and Benjamini, Krieger and Yekutiely tests (*p* = 0.89). In total, 44 families were identified (Supplementary Figure S2 and Supplementary Table S2). In individuals from the Buiuçu and Puruzinho communities the most abundant families were *Prevotellaceae* (86 OTUs and 237,670 sequences, and 86 OTUs and 269,197 sequences, respectively; Supplementary Tables S2a,b) and *Ruminococcaceae* (281 OTUs and 179,255 sequences, and 277 OTUs and 230,093 sequences, respectively; Supplementary Tables S2a,b). Among the individuals from Rio de Janeiro the most abundant families were *Bacteroidaceae* (122 OTUs and 333,159 sequences) and *Ruminococcaceae* (271 OTUs and 176,623 sequences) (Supplementary Table S2c). Thus, the relative abundance of *Bacteroidaceae* and *Prevotellaceae* families differentiated communities of the two geographical regions. As with the family analysis described above, the relative abundance of bacterial genera showed differences between the community of Rio de Janeiro and the Amazon, with the identification of 76 genera (Figure 2 and Supplementary Table S2). In the Buiuçu community samples, the most abundant genera were *Prevotella* (86 OTUs and 237,670 sequences), *Dialister* (22 OTUs and 62,650 sequences), and unidentified members of the *Ruminococcaceae* (216 OTUs and 120,253 sequences) and *Succinivibrionaceae* families (18 OTUs and 58,027 sequences) (Supplementary Table S2a). The most abundant genera in individuals from the Puruzinho community were *Prevotella* (86 OTUs and 269,197 sequences), *Dialister* (22 OTUs and 110,161 sequences), and unidentified members of the *Ruminococcaceae* family (214 OTUs and 155,893 sequences) and the *Clostridia* class (88 OTUs and 55,599 sequences) (Supplementary Table S2b). In subjects from Rio de Janeiro the most abundant genera were *Bacteroides* (112 OTUs and 333,159 sequences), unidentified members of the *Lachnospiraceae* family (293 OTUs and 148,669 sequences), and unidentified members of the *Clostridia* class (96 OTUs and 62,668 sequences) (Supplementary Table S2c). Therefore, the relative abundance of the *Bacteroides* and *Prevotella* genera differentiated the composition of the intestinal bacteriome of individuals living in the Amazon region and Rio de Janeiro, although the difference in composition between the three communities at the genus level was not statistically significant (Kruskal-Wallis and Benjamini, Krieger and Yekutiely tests; *p* = 0.69). Nevertheless, we performed pattern analyses using Pearson r as the distance measure and found that levels of the two most discriminating genera between Amazon versus Rio de Janeiro samples, Bacteroides and Prevotella, were significantly different, as shown in Figure 3.

In order to assess how the bacterial communities found in individuals from the three locations relate to each other, we constructed Venn diagrams to get insights into the OTUs that were shared among the three investigated communities or exclusively associated with each one of them (Supplementary Figure S3). Samples from all subjects studied, from the three communities, shared 893 out of the 1,096 identified OTUs, confirming that there is a large bacterial core in the human intestinal microbiota and that strong selective forces shape these bacterial communities in similar ways in populations that are distinct both geographically as well as in terms of lifestyle. This core is dominated by OTUs affiliated with *Prevotella* (69 OTUs and 539,780 sequences), a genus of the *Ruminococcaceae* family (206 OTUs and 370,915 sequences), *Bacteroides* (69 OTUs and 312,153 sequences), and *Dialister* (21 OTU and 188,757 sequences) (Supplementary Table S3a). However, some OTUs were exclusively found in one of the communities studied. Samples from the Buiuçu and the Puruzinho communities had 14 and three specific OTUs, respectively, whereas individuals from Rio de Janeiro displayed 52 specific OTUs (Supplementary Figure S3). The Buiuçu community was dominated by *Treponema* (1 OTU and 14,191 sequences, Supplementary Table S3b), whereas the most abundant member of the Puruzinho community is a genus of the *Paraprevotellaceae* family (1 OTU and 790 sequences, Supplementary Table S3c). Individuals from Rio de Janeiro were dominated by *Bacteroides* (1 OTU and 18,322 sequences, Supplementary Table S3d). These data show that although the level of specificity was low, a few OTUs do provide a clear distinction between the samples from Rio de Janeiro and those from individuals living in the Amazon region when numbers of community-specific OTUs are observed.

**FIGURE 4:**
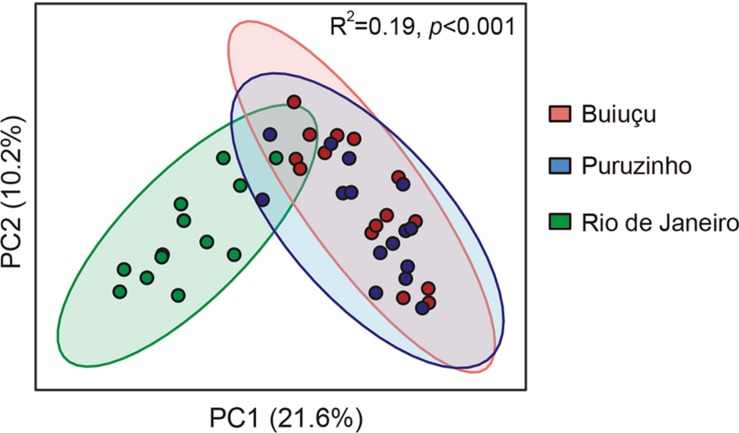
Principal coordinate analysis of unweighted UniFrac distances showing beta diversity of the gut microbiome of individuals belonging to the three communities studied. Green circles represent subjects from Rio de Janeiro, red circles are subjects from Buiugu and blue represents subjects from Puruzinho. R2 and *p* value were calculated using ANOSIM.

We then performed PCoA analyses on the results obtained to display beta diversity in the total bacterial communities of the individuals studied (Figure 4). The total variation found in the gut bacteriome was explained in 31.8% by the unweighted UniFrac analysis; the PC1 axis had the largest contribution, accounting for 21.6% of the variation found in the intestinal bacteriome, and axis 2 accounted for 10.2% of the discrimination. Subjects from Rio de Janeiro formed a well-defined group that could be easily distinguished from the other two groups based on the PCoA. Individuals from the Puruzinho and Buiuçu communities did not form very well-defined groups, and there was a major overlap between samples from each of these groups in our PCoA analysis (Figure 4). Statistical analysis using ANOSIM showed that the PCoA profiles were significantly different (*R*^2^ = 0.19; *p* < 0.001). These results were corroborated when we performed statistical analyses on comparisons of averaged relative abundances of OTUs between the three sample groups. We first tested our dataset to determine its distribution using both the D’Agostino & Pearson as well as the Shapiro-Wilk normality tests. Both tests showed that the data does not follow a normal (Gaussian) distribution (*p* < 0.0001 for all sample groups in both tests). We then compared OTU abundance distribution between sample groups using the Kruskal-Wallis test with multiple comparison FDR correction using the Benjamini, Krieger and Yekutiely test. Such analysis showed that OTU abundance of both Puruzinho and Buiuçu samples were significantly different from that found in samples from Rio de Janeiro (*p* = 0.0013 and *p* = 0.0014, respectively). In contrast, a comparison between OTUs found in Puruzinho and Buiuçu samples did not reveal statistically significant differences (*p* = 0.98). Based on these results, it is clear that the gut bacteriome of individuals from Amazon populations is highly divergent from that of subjects living in an urban environment. However, we were not able to clearly differentiate individuals from the two Amazon communities based on their gut bacteriome composition.

After assessing the microbial composition of the intestinal tract of individuals from the three communities studied, we set out to investigate if the metabolic composition of the intestinal tract varied among these subjects. We have previously used metabolomics to study the chemical composition of murine feces and showed that thousands of small molecules are present and that the gut microbiome is a major determinant of fecal chemical composition (Antunes et al., 2011b). To assess the fecal metabolome of individuals from the three groups studied herein, we extracted small molecules from fecal samples and studied their chemical composition through DI-FTICR-MS, as previously described (Antunes et al., 2011a, b). We detected a total of 7,210 metabolite features, with 3,469 detected in the negative-ion mode and 3,741 in the positive-ion mode. We then filtered this dataset to include only metabolites that were present in at least *n*-1 samples (four samples in this case) of at least one of the sample groups (Puruzinho, Buiuçu and Rio de Janeiro). By doing so, we detected a total of 4,745 metabolite features, with 2,071 detected in the negative-ion mode and 2,674 in the positive-ion mode. The number of metabolite features that fulfilled this filtering criterion in each of the sample groups varied between 1,476 and 2,106, as can be seen in Table 2.

**TABLE 2:** Summary of DI-FTICR-MS results.

	**Negative ESI^a^**	**Positive ESI^a^**	**Total**
**Ions detected**			
Number of ions	3469	3741	7210
Minimum replicates^b^	2071	2674	4745
B^c^	1627	1476	3103
P^c^	1787	2106	3893
R^c^	1634	1761	3395

**Ions affected ≥ 2-fold^d^**			
B versus P	213	457	670
B versus C	117	243	360
P versus C	335	771	1106

Total		2136

*^*a*^ESI, electrospray ionization. ^*b*^Ions detected in a minimum of *n-*1 samples from at least one of the three sample groups. ^*c*^B, Buiuçu; P, Puruzinho; R, Rio de Janeiro. ^*d*^*p* value <0.05, as determined by unpaired *t* tests.*

**FIGURE 5:**
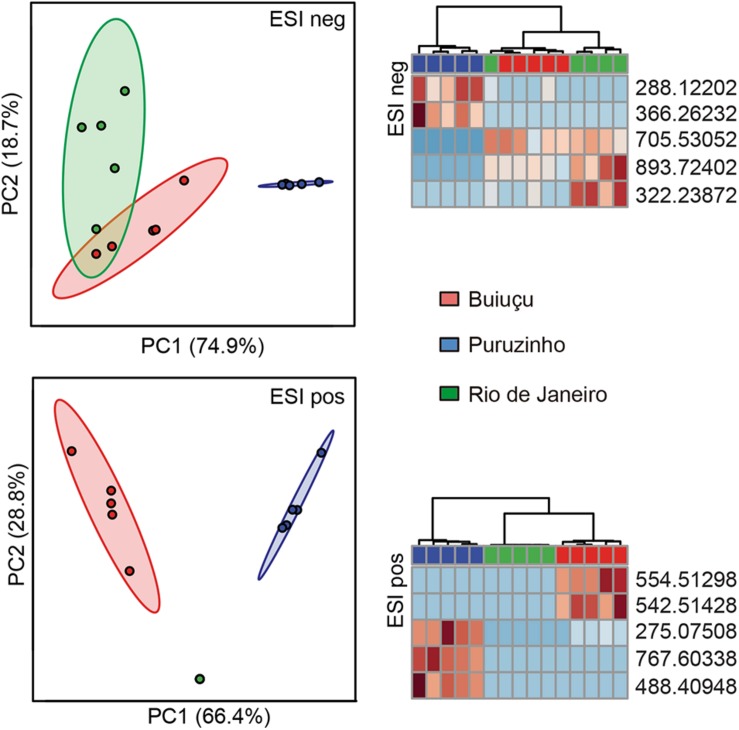
Principal component analysis, heat maps and dendrograms of the 5 top ions from DI-FTICR-MS data. The top 5 ions regarding their discriminatory power were selected and PCA plots and heat maps were constructed based on their intensities. Only ions that were present in a minimum of *n*-1 replicates of at least one of the three sample groups were included. Data for negative (ESI neg) and positive (ESI pos) ionization modes are shown. In PCA, some of the data points can not be clearly seen due to overlapping (*n* = 5 in all groups). In heat maps each column represents one sample, with the top rectangle indicating the source of the sample: red rectangles represent samples from the Buiuçu community, whereas blue rectangles are for Puruzinho samples and green are for Rio de Janeiro samples. Each line in the heat maps represent one ion, and colors indicate levels of each ion compared to the intra-sample average. Red indicates ions detected at levels higher than the average whereas blue represents ions that were found in levels lower than the average.

Next, we probed the total set of metabolites filtered above to determine if there were significant changes in metabolite composition between the sample groups. To do so, we used the Statistical Analysis tool of the online software MetaboAnalyst to construct PCA plots comparing samples from the Buiuçu, Puruzinho, and Rio de Janeiro communities. Supplementary Figure S4 shows that, when using the totality of metabolites found in *n*-1 samples, there were no observable differences between the metabolic profiles of the three sample groups. We then constructed heat maps and dendrograms based on these data and found an observable, although only partial, separation of samples from the Puruzinho community from the 2 other communities (Supplementary Figure S5). Although these observations show that the metabolic profile as a whole cannot be used to discriminate between the three sample groups, we asked whether there would be a subset of metabolites that could be somewhat specific to each of the communities studied. We constructed heat maps and dendrograms using only the top 25 metabolites that showed a higher discriminatory power, as determined by ANOVA. As can be seen in Supplementary Figure S6, by doing so we were able to fully discriminate samples from the three groups when using the positive ionization mode data. Data from negative ionization was also able to discriminate groups, with the exception of one sample from the Rio de Janeiro community that grouped with samples from the Buiuçu community. The same results could be obtained when using as few as five metabolites per ionization mode, with a complete separation between the three groups in the positive ionization mode data and partial discrimination when using the negative ionization data, as can be seen in PCA, heat map, and dendrogram analyses (Figure 5). Since the individuals living in the Buiuçu and Puruzinho communities are exposed to similar environmental conditions, which are distinct from those found in Rio de Janeiro, we performed PCA, heat map, and dendrogram analyses of our data using only 2 groups, namely Amazon versus Rio de Janeiro samples. PCA, heat map, and dendrogram plots showed no observable differences when all ions detected in negative ionization mode were considered (data not shown). However, when using positive ionization data, a clear separation between samples from the Amazon and Rio de Janeiro could be obtained in heat map and dendrogram analyses (Supplementary Figures S4, S5). We then constructed dendrograms and heat maps using the top 25 and 5 metabolites with the highest discriminatory power using data from positive ionization mode. Supplementary Figures S6, S7 show that these analyses allowed us to clearly differentiate Amazon and Rio de Janeiro samples.

**FIGURE 6:**
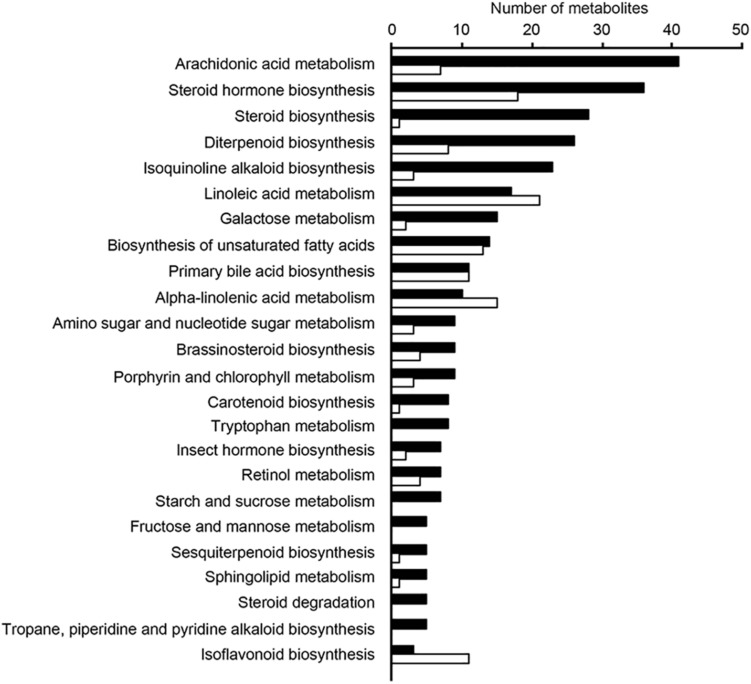
Pathways with differences in metabolite levels between the Amazon and Rio de Janeiro sample groups. Ions showing differences of at least 2-fold in relative intensity values between these groups were assigned to KEGG pathways using the Pathos online software. This was done for both positive (black bars) and negative (white bars) ionization data. Bars represent the number of assigned metabolites in each pathway. Only pathways that had at least 5 assigned ions are shown.

In addition, relative levels of the 5 metabolites with the highest discriminatory power (in positive ionization mode) were compared between these two samples groups, and Supplementary Figure S7 shows that the group to which samples belonged could be easily predicted using these data. We then assessed the relative levels of the ten metabolites with the highest discriminatory power (in both negative and positive ionization modes) in the three sample groups and found that levels of individual ions were also highly associated with specific sample groups, as shown in Supplementary Figure S8. We next attempted to map the ten metabolites from above to KEGG metabolic pathways, using the Pathos online tool. None of the five metabolites from the negative ionization data mapped to any KEGG pathways. When using the five metabolites found in the positive ionization data, only one of them mapped to a KEGG pathway; *m/z* 275.07508 was putatively identified as deoxyinosine, mapping to the purine metabolism pathway. In order to investigate the metabolic pathways that differed between samples from the three communities studied, we calculated fold-differences in relative metabolite levels and mapped metabolites showing at least 2-fold differences in abundance to KEGG pathways using Pathos. Initially, we mapped metabolites whose levels differed between the combined group of Amazon samples (Buiuçu and Puruzinho) versus Rio de Janeiro samples, and the results of such comparison are shown in Figure 6. As can be seen from these data, the metabolic pathway showing the highest number of metabolites differing between the Amazon and Rio de Janeiro sample groups was that of arachidonic acid metabolism. We also performed binary comparisons, and mapped metabolites whose levels differed when comparing each of the three sample groups (Buiuçu, Puruzinho, and Rio de Janeiro) against each other. The results of these analyses are shown in Supplementary Figures S9–S11.

## Discussion

Classical studies of the gut microbiome were dependent on microbial cultivation techniques. However, the use of molecular techniques revealed that a significant portion of the gut microbiome cannot be cultured using standard microbiological methods. As a consequence, much of the metabolic diversity of microorganisms present in the human gut microbiome remains unknown (Browne et al., 2016; Ito et al., 2018). More recently, the development and use of high-throughput sequencing technologies has revealed that the intestinal microbiota is far more complex and diverse than previously thought (Qin et al., 2010; Human Microbiome Project Consortium, 2012). Additionally, the impact of external factors on gut microbial composition has been studied, and work has been conducted to assess the connection between eating habits and intestinal microbial profiles (Segata, 2015; Chen et al., 2016; Nishijima et al., 2016; Zopf et al., 2018). In this study, we used high-throughput 16S rDNA sequencing of feces to study bacterial composition and predict functionality. The high number of OTUs observed (1,096) showcases the great bacterial diversity found in the intestinal microbiome of the study subjects and is in line with common wisdom in the field (Human Microbiome Project Consortium, 2012). Alpha diversity analysis of these bacterial communities was estimated by the Shannon diversity index, which showed that all samples are substantially diverse, and that the greatest level of diversity was found among samples from individuals living in Rio de Janeiro, a highly populated and industrialized area. We hypothesize that this is due to the fact that these individuals have a varied diet when compared with individuals from the two Amazonian communities, since individuals living in an urban environment are exposed to a wider variety of food sources. However, the difference in Shannon index between individuals from the three communities was not statistically significant.

Although there were no major differences in microbiome structure between samples from the two Amazon populations, the relative abundance of some representative OTUs showed clear differences when the intestinal microbiome from individuals of Amazon communities was compared to that of individuals from Rio de Janeiro. When relative abundance values were calculated and compared at the phylum level we were not able to distinguish between the three communities, and these results are in line with the general knowledge in the field that few phyla make up the human intestinal microbiota and that there is a low inter-individual variation at the phyla level. Among the 12 phyla identified in this study, *Firmicutes* and *Bacteroidetes* were the most abundant ones in all samples. This is in agreement with bacterial profiles commonly detected in the human gut and reported in the literature. On the other hand, the relative abundance of bacterial families revealed observable differences between the communities of Rio de Janeiro and those in the Amazon. Individuals from Rio de Janeiro have a bacterial community predominantly composed of *Bacteroidaceae* and *Ruminococcaceae* families, together representing more than 50% of all the diversity of families found. Individuals of the Puruzinho and Buiuçu communities, on the other hand, had *Prevotellaceae* and *Ruminococcaceae* as the most abundant families, indicating that variations in community structure can be found at lower taxonomic levels. Although this difference was not statistically significant, we believe this is due to the relatively small number of samples analyzed, which limits the statistical power for detecting significant differences. Similarly, the relative abundance of bacterial genera showed observable, though not statistically significant, differences when the community composition of samples from Rio de Janeiro and from the Amazon were compared. Individuals from Rio de Janeiro showed higher abundance of *Bacteroides*, *Faecalibacterium* and of unclassified bacteria belonging to the *Ruminococcaceae* family and the *Clostridiales* order. By themselves, these taxa represent around 55% of all bacterial diversity. In the individuals from the Puruzinho community, the intestinal microbiota is mainly composed of *Prevotella*, *Dialister*, and unclassified members of the *Ruminococcaceae* family and the *Clostridiales* order. These four taxa represent about 55% of the intestinal bacterial composition. At last, among the individuals of the Buiuçu community, the most abundant genera were *Prevotella, Dialister*, and unclassified members of the *Ruminococcaceae* and *Enterobacteriaceae* families. These taxa comprise about 50% of the intestinal bacteriome of these individuals. Thus, the community of Rio de Janeiro and the Amazon communities are identified by the prevalence of *Bacteroides* and *Prevotella*, respectively. This observation is in line with the current model on the effect of diet on the gut microbiome, where individuals with a more varied and high-protein diet (which is expected of individuals from western urban settings) have their intestinal microbiota dominated by the *Bacteroides* genus, whereas people with a diet rich in vegetable fibers and carbohydrates (which is observed in individuals from Amazonian communities) have their intestinal microbiota dominated by *Prevotella* (Wu et al., 2011). While studying the gut microbiomes of coexisting hunter-gatherer and agricultural communities in the Central African Republic, Gomez et al. (2016) showed that higher relative levels of *Prevotellaceae* are significant markers of microbiomes from the hunter-gatherer community. Interestingly, the authors also analyzed the microbiome of western individuals (United States) using data from the Human Microbiome Project (Human Microbiome Project Consortium, 2012) and found that the microbiome of the agriculturalist Bantu community displayed western-like features. Such pattern was also observed by Dhakan et al. (2019), who studied the gut microbiome composition of communities living in geographically distinct areas of India, and showed that individuals who consumed a primarily plant-based diet had significantly higher levels of *Prevotella* in their microbiomes, in contrast to microbiomes of individuals living in omnivorous communities, who displayed higher levels of *Bacteroides*, *Ruminococcus*, and *Faecalibacterium* (Dhakan et al., 2019). Although we believe that these observations are in line with our findings, it is important to note that a careful analysis of dietary patterns of the subjects participating in our study was not performed, and this would be necessary in order to fully confirm this diet-microbiome association. Interestingly, though, the same profile of bacterial dominance shown here was found in another study, by Clemente et al. (2015), investigating the intestinal microbiota of individuals from an indigenous tribe of the Venezuelan Amazon. It is also noteworthy that we found a higher abundance of the *Dialister* genus in the bacteriome of individuals of the two Amazonian communities, when compared to individuals from Rio de Janeiro. Although, we cannot ascertain the exact reason for this, it may be related to the poor hygiene conditions found in populations living in the Amazon, where living conditions are precarious. Members of this genus are responsible for several diseases in humans, including periodontitis, ulcerative gingivitis, respiratory infections, brain abscesses, and bacteremia (Morio et al., 2007).

Using Venn diagrams, we verified that individuals of the three communities share most of the OTUs identified, which demonstrates that a large portion of the identified OTUs are shared by the intestinal bacteriome of all individuals analyzed. We suggest that these OTUs are part of the bacterial core of the human gut microbiome, composed mainly by *Prevotella*, a genus of the family *Ruminococcaceae*, *Bacteroides*, and *Dialister*. On the other hand, we also found that three, 14, and 52 OTUs are unique to individuals from Puruzinho, Buiuçu, and Rio de Janeiro, respectively. Among the exclusive OTUs, the most abundant was *Treponema* for the Buiuçu community, a genus of the *Paraprevotellaceae* family for Puruzinho community, and *Bacteroides* for Rio de Janeiro. These results confirm the higher alpha diversity found among individuals representing an urban population with varied diet and show that only a small number of OTUs is unique to each community. Also, the data show a striking feature of the human intestinal microbiota, which is the existence of a bacterial core found in all individuals. Previous studies in the literature described this core microbiome; knowledge in this field draws attention to the fact that the microbiota-host relationship is finely controlled and balanced, and therefore requires (or is the cause of) a certain degree of stability (Tschöp et al., 2009; Turnbaugh et al., 2009; Qin et al., 2010; Aguirre de Cárc, 2018).

Nevertheless, PCoA on DNA sequencing data showed a clear distinction between gut bacterial communities from individuals living in an industrialized setting when compared to communities from individuals living in the Amazon. However, no significant separation was observed when the communities from the two Amazon populations were compared. This finding supports the hypothesis that diet and lifestyle are major factors driving gut microbiome composition. Nevertheless, it is clear that multiple factors influence microbial composition in the gut and that the complete elucidation of how these factors integrate is challenging at best. The two Amazonian communities have marked environmental and lifestyle differences, but these differences alone did not result in distinct gut bacterial communities. At least to some extent, the gut bacteriome of these individuals reflects the unique conditions they live in, with the bacteriome from subjects living in an industrialized city differing markedly from those living in the Amazon.

Results from metabolomics analyses showed that although the general metabolic profile of samples did not display marked differences that allowed us to distinguish each of the three sample groups, a subset of metabolites could be used to successfully predict the origin of the samples. Additionally, thousands of metabolites showed relative levels with at least 2-fold differences when binary comparisons between the sample groups were performed. Previous work from our group has shown that the human gastrointestinal tract is a rich source of chemical diversity, with thousands of metabolites present, and that this chemical diversity can be readily studied through high-throughput metabolomics (Antunes et al., 2011a, b). Our studies have shown that the gut microbiome is a critical determinant of the chemical composition of the gut lumen, and that treatments that disturb this microbial ecosystem have profound effects on the human gut metabolome (Antunes et al., 2011a, b). In line with these previous studies, our current data shows that differences in the gut metabolome of individuals with marked lifestyle differences can be found. Although the changes reported herein are not nearly as dramatic as those found during antibiotic treatment or enteric infection, this is to be expected, since dietary changes are unlikely to affect gut microbial composition to the same extent as these two other interventions (Antunes et al., 2011a, b). It is also interesting to note that, although with different extents, the changes observed in the present study share resemblance with those observed after antibiotic treatment and enteric infection (Antunes and Finlay, 2011; Antunes et al., 2011a, b). In the present study, among the metabolites whose levels differed between sample groups and for which putative identities could be obtained in databases, the most abundantly detected metabolic pathway was that of arachidonic acid metabolism. This metabolic pathway was also found to be responsive to antibiotic treatment and *Salmonella* infection (Antunes et al., 2011a, b). Although we cannot rule out that our extraction and analyses methods favored the detection of arachidonic acid metabolites, this alone could not account for the differences observed. In our previous studies cited above, although arachidonic acid was one of the pathways modulated by antibiotic treatment and infection, it was not the main pathway affected, as was the case in the current study. Additionally, previous experiments using an alternative method (enzyme-linked immunosorbent assays) confirmed the modulation of a few arachidonic acid metabolites by the gut microbiome (Antunes et al., 2011a, b). In summary, we showed that two communities living in remote areas of the Amazon display marked changes in gut microbial and metabolic compositions when compared to individuals from an urban environment. At least some of the metabolic pathways that differ between the groups are critical for the maintenance of gut homeostasis, as is the case of the eicosanoids derived from arachidonic acid (Wallace, 2018). We have previously shown that one metabolite from this pathway, 15-deoxy-Δ^12,14^-prostaglandin J_2_, may play an important role in the defence against infection (Buckner et al., 2013), and have also described the potential role of metabolites produced by the gut microbiome in the resistance to pathogen colonization (Antunes et al., 2010, 2014; Peixoto et al., 2017). Whether the gut metabolic changes described here in populations from the Amazon may result in different susceptibility profiles to enteric infections and other diseases remains to be determined. To the best of our knowledge, this is the first report of the characterization of the gut microbiome of Amazon populations and its associated metabolome.

## Data Availability

The datasets generated for this study can be found in National Center for Biotechnology Information (NCBI) Sequence Read Archive (SRA), PRJNA547608.

## Ethics Statement

This study was carried out in accordance with the recommendations of the Research Ethics Committee of the Institute for Studies of Collective Health of the Federal University of Rio de Janeiro, with written informed consent from all subjects. All subjects gave written informed consent in accordance with the Declaration of Helsinki. The protocol was approved by the aforementioned Research Ethics Committee.

## Author Contributions

EP, KM, and DS designed the study, performed the experiments, and collected the data. CH designed the study, collected and analyzed the data, and wrote the manuscript. LL, RD, and LA designed the study, collected and analyzed the data, provided the reagents, and wrote the manuscript. DdC collected and analyzed the data, and provided the reagents. JH performed the experiments, collected and analyzed the data, and provided the reagents and analytical tools. CB analyzed the data, and provided the reagents and analytical tools. RF collected and analyzed the data, and wrote the manuscript. JS collected and analyzed the data, and provided the reagents and analytical tools.

## Conflict of Interest Statement

The authors declare that the research was conducted in the absence of any commercial or financial relationships that could be construed as a potential conflict of interest.

## Footnotes

1 http://ngs.igsb.anl.gov

2 http://www.microbiomeanalyst.ca

3 http://www.metaboanalyst.ca

4 http://motif.gla.ac.uk/Pathos/
