# Supplementary figures and images for: The Gut Microbiome and Metabolome of Two Riparian Communities in the Amazon
Source: Front Microbiol. 2019 Sep 4;10:2003. doi: 10.3389/fmicb.2019.02003 (PMC6737013; doi:10.3389/fmicb.2019.02003)

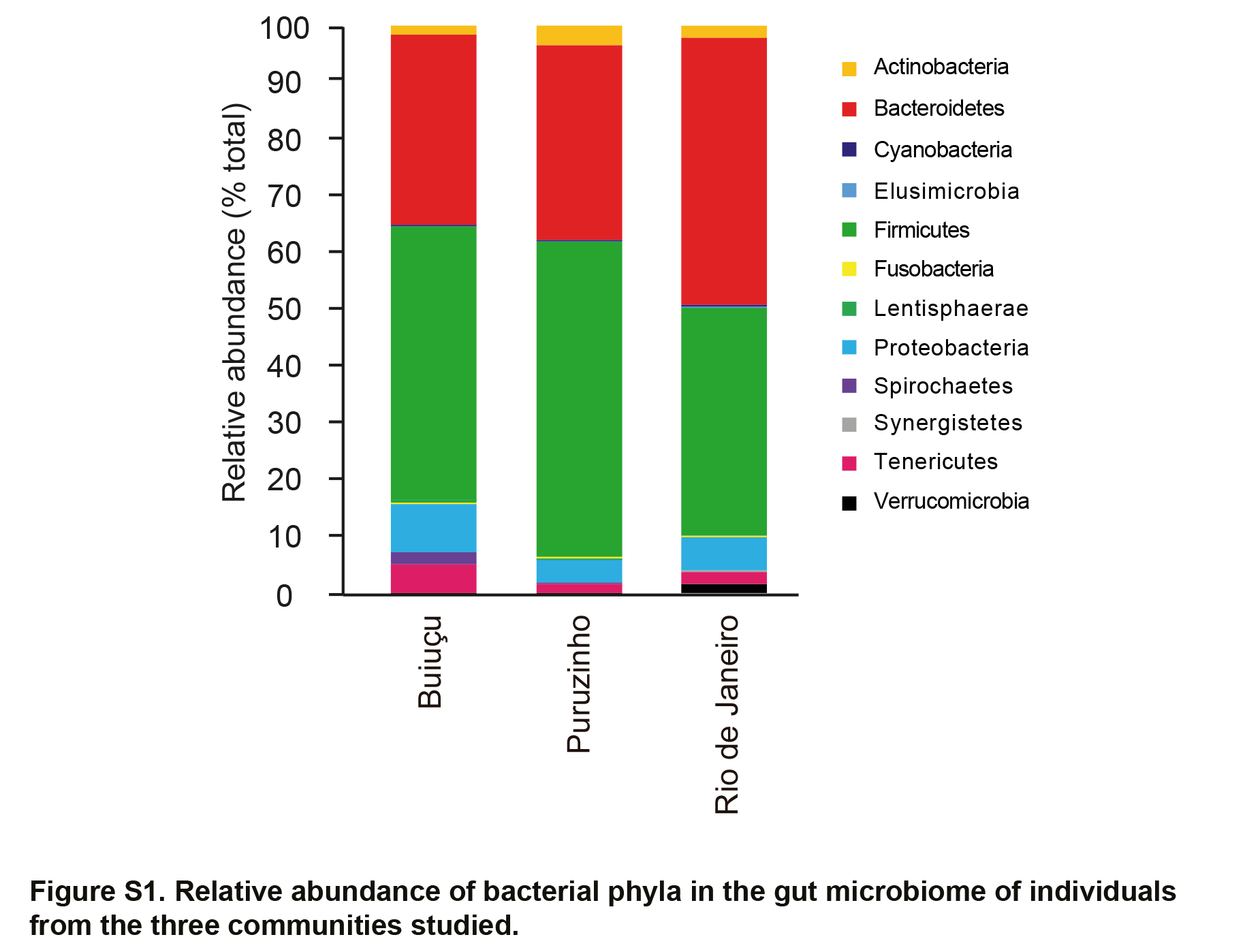

Supplement: Supplementary file 1 [file Image_1.JPEG]

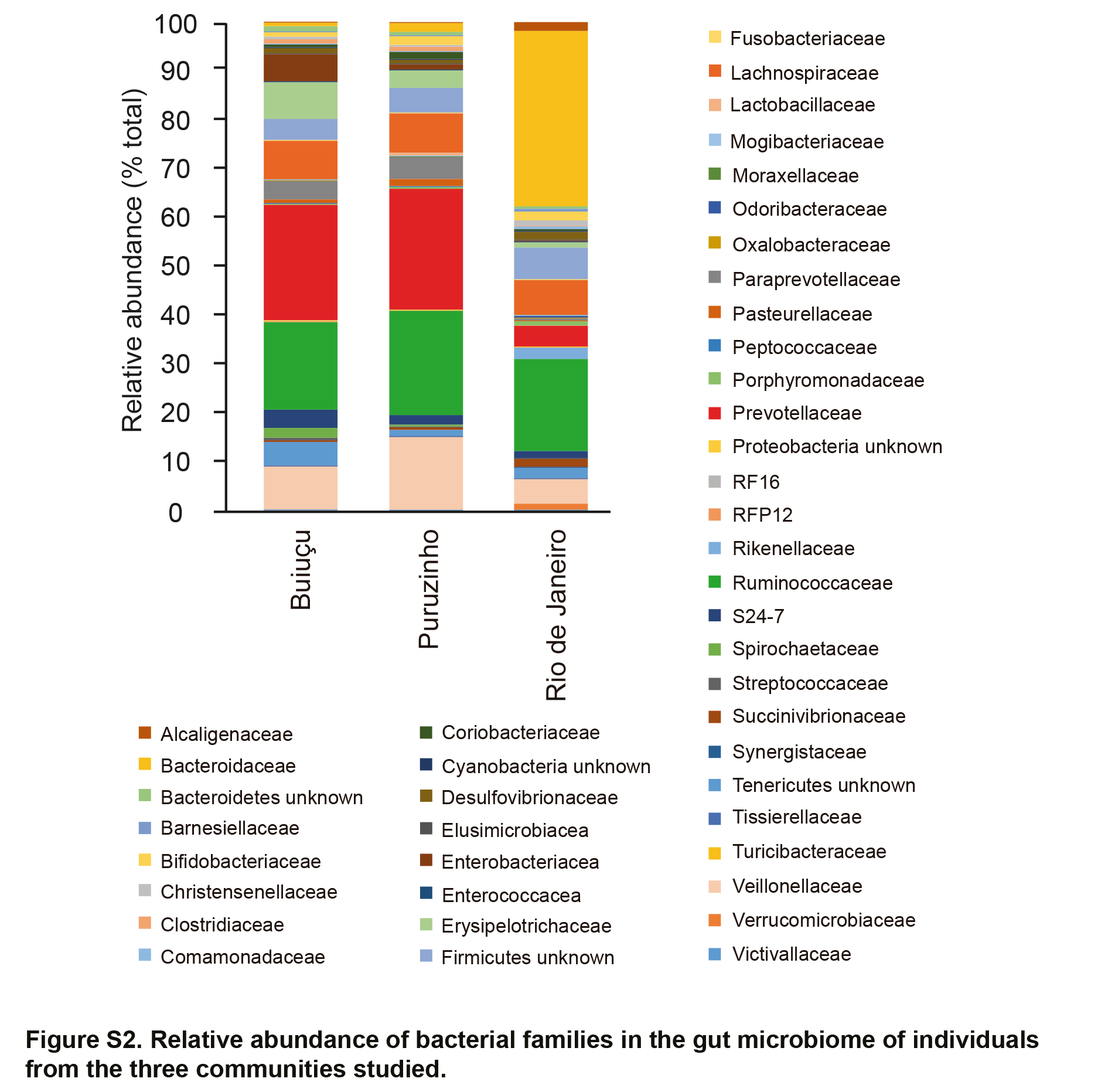

Supplement: Supplementary file 2 [file Image_2.JPEG]

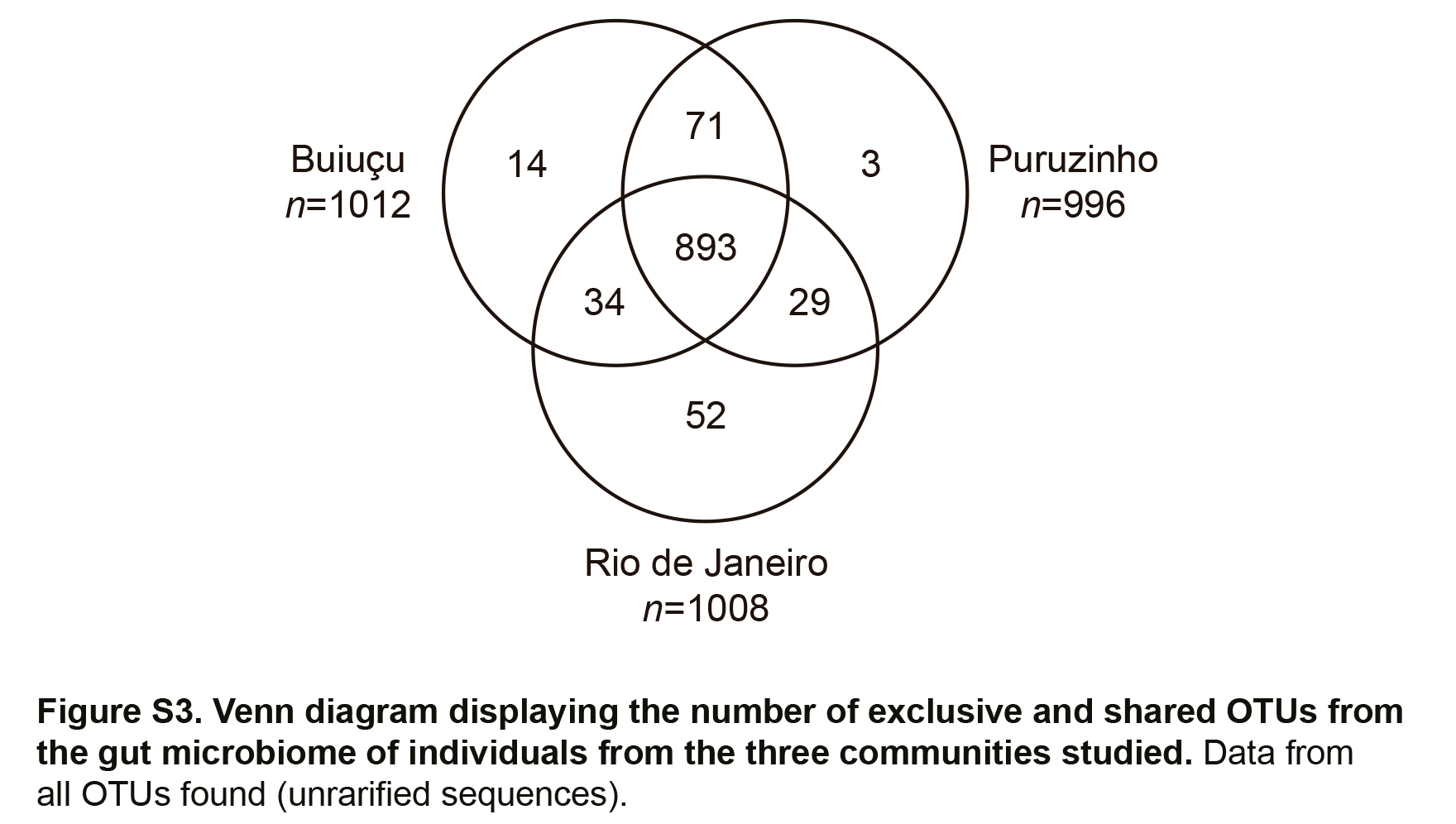

Supplement: Supplementary file 3 [file Image_3.JPEG]

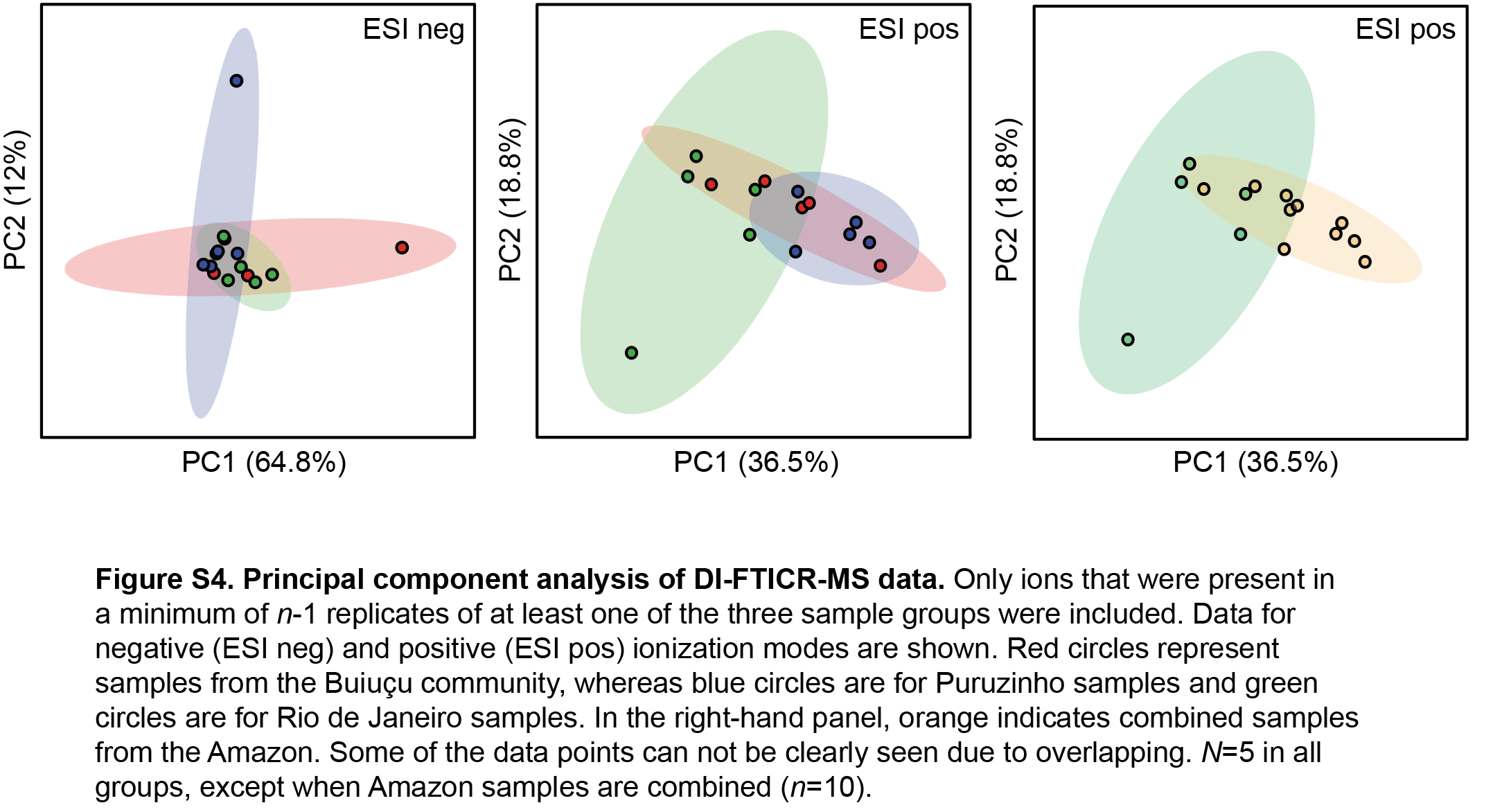

Supplement: Supplementary file 4 [file Image_4.JPEG]

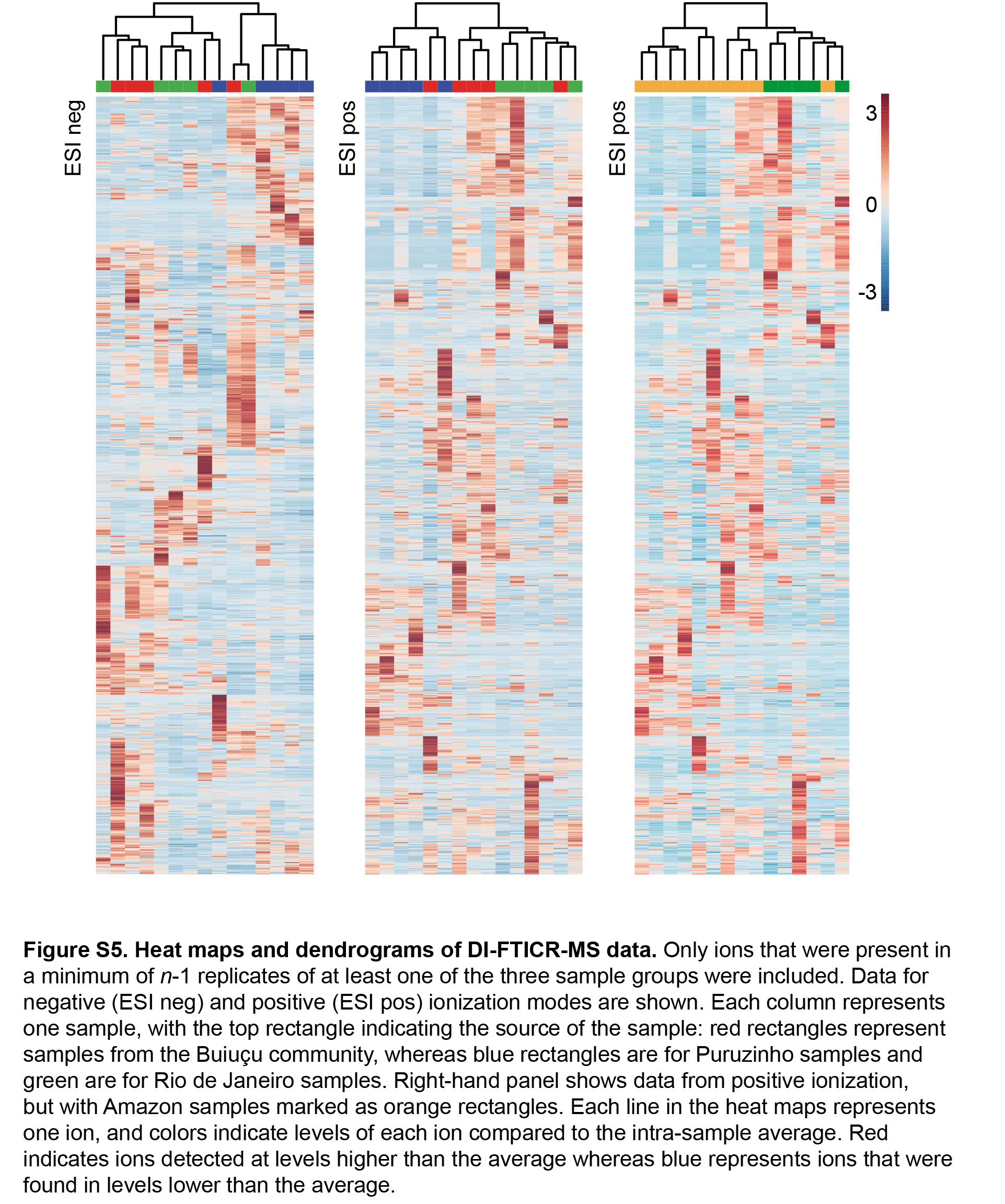

Supplement: Supplementary file 5 [file Image_5.JPEG]

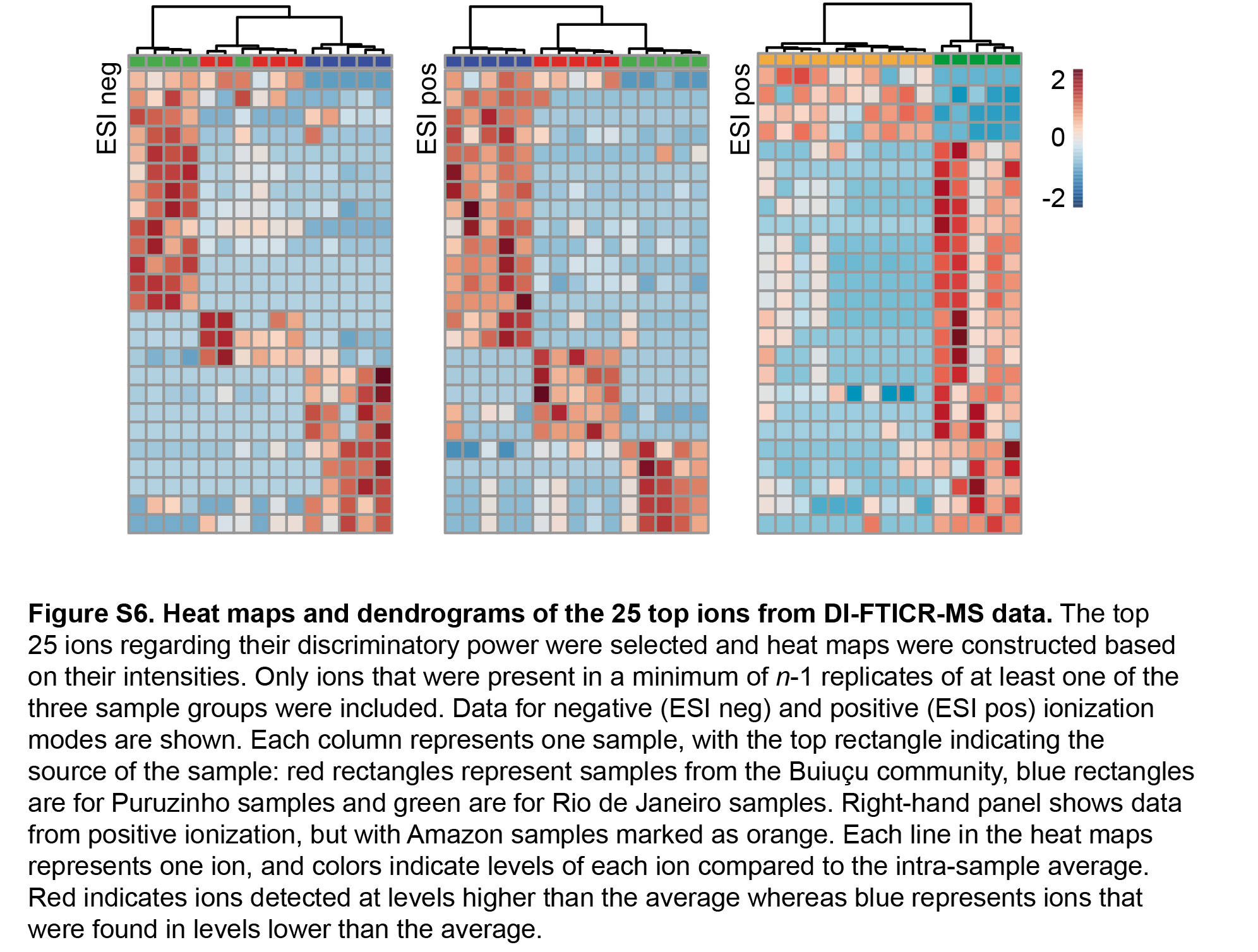

Supplement: Supplementary file 6 [file Image_6.JPEG]

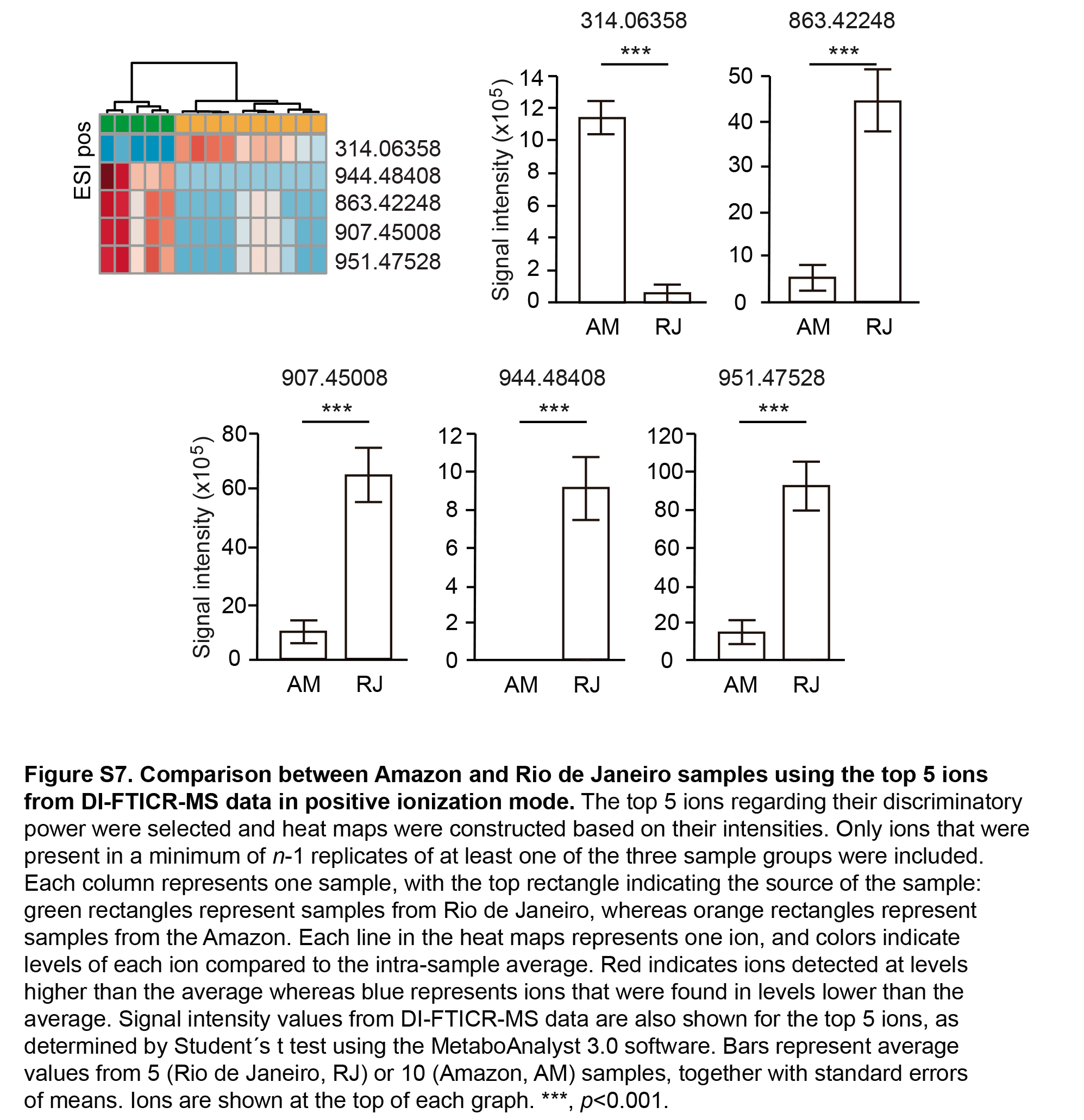

Supplement: Supplementary file 7 [file Image_7.JPEG]

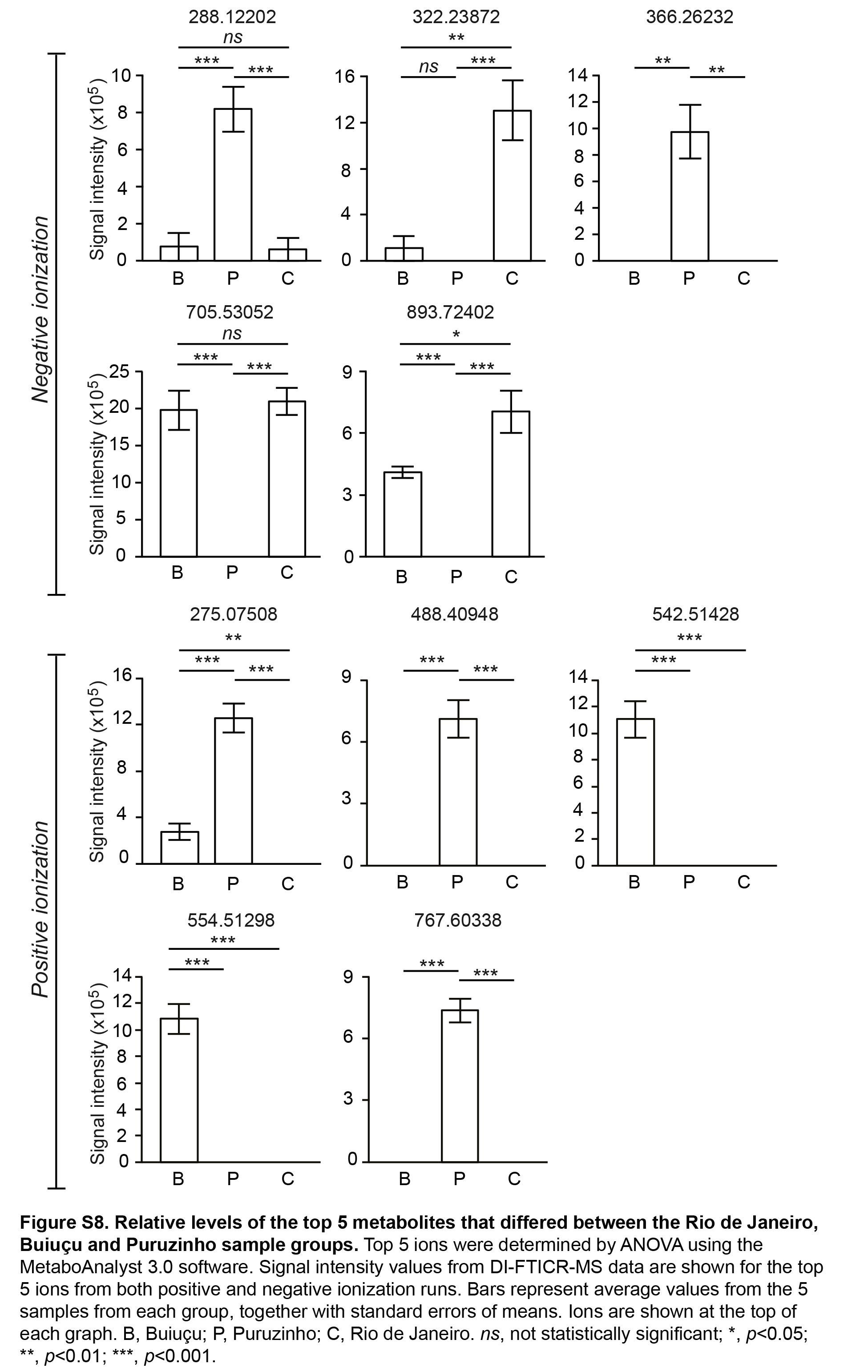

Supplement: Supplementary file 8 [file Image_8.JPEG]

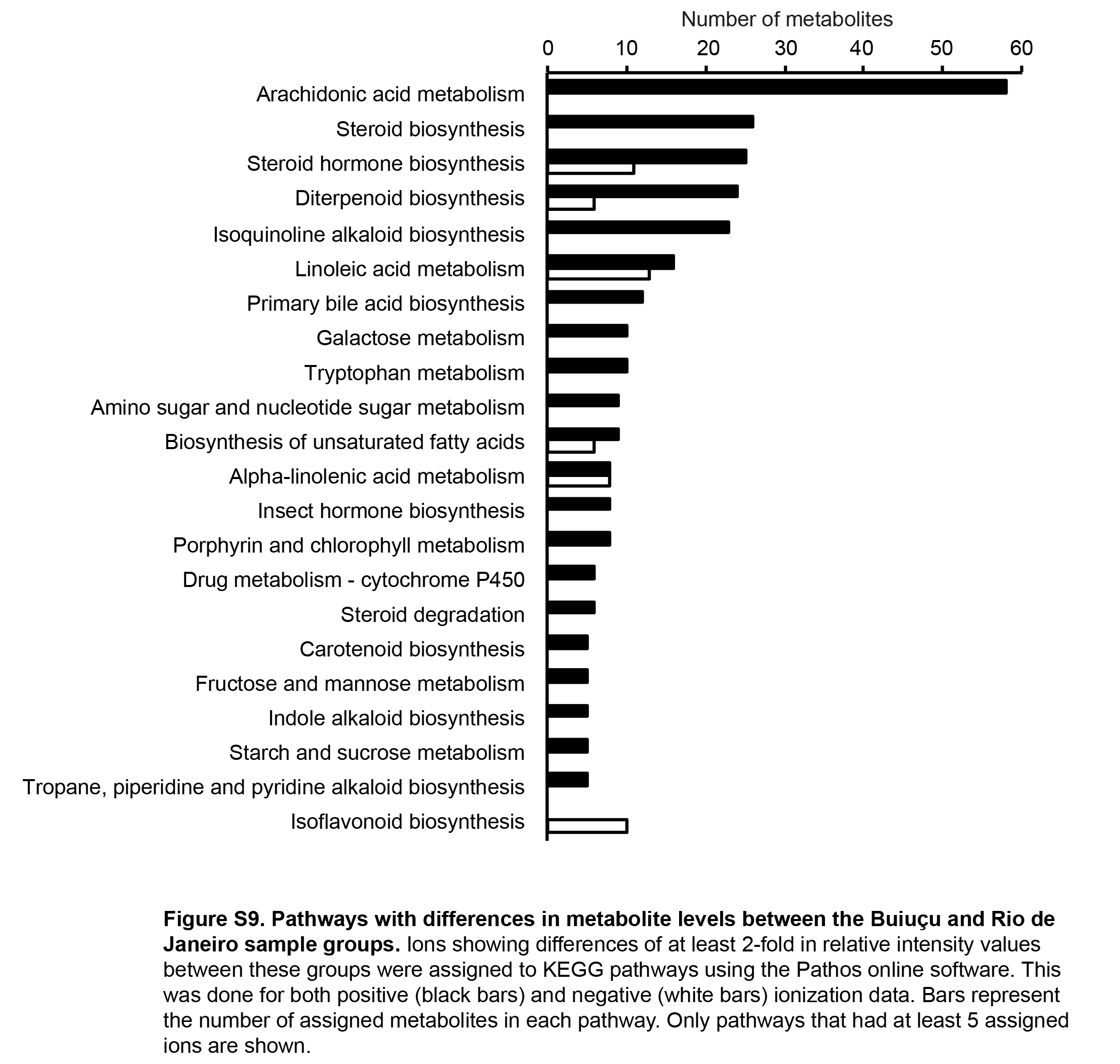

Supplement: Supplementary file 9 [file Image_9.JPEG]

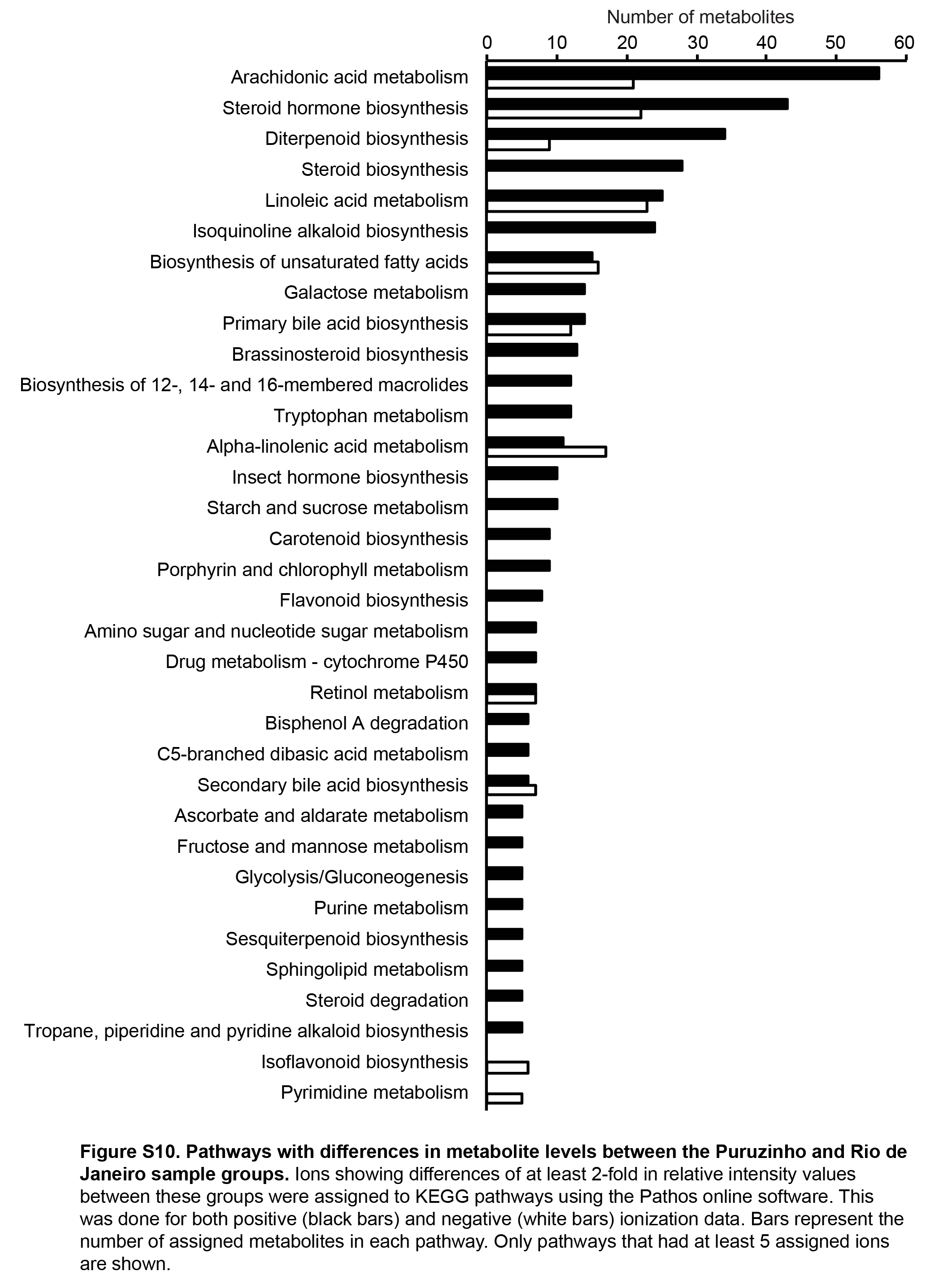

Supplement: Supplementary file 10 [file Image_10.JPEG]

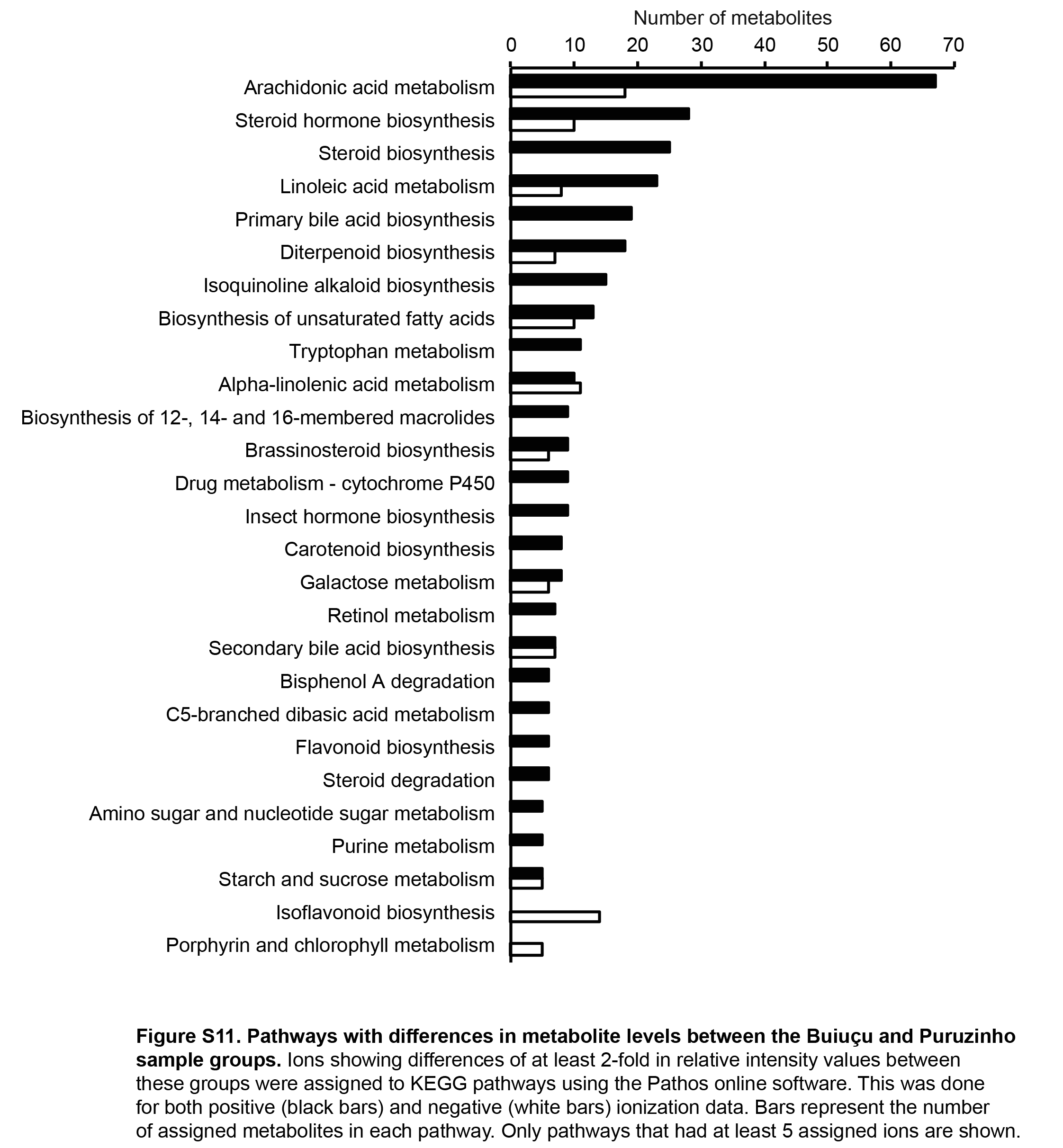

Supplement: Supplementary file 11 [file Image_11.JPEG]
